# Microsatellite Instability, KRAS Mutations and Cellular Distribution of TRAIL-Receptors in Early Stage Colorectal Cancer

**DOI:** 10.1371/journal.pone.0051654

**Published:** 2012-12-20

**Authors:** Lydia Kriegl, Andreas Jung, David Horst, Antonia Rizzani, Rene Jackstadt, Heiko Hermeking, Eike Gallmeier, Alexander L. Gerbes, Thomas Kirchner, Burkhard Göke, Enrico N. De Toni

**Affiliations:** 1 Institute of Pathology, University of Munich, Munich, Germany; 2 Department of Medicine II, University Hospital Grosshadern, University of Munich, Munich, Germany; The University of Kansas Medical Center, United States of America

## Abstract

**Background:**

The fact that the receptors for the TNF-related apoptosis inducing ligand (TRAIL) are almost invariably expressed in colorectal cancer (CRC) represents the rationale for the employment of TRAIL-receptors targeting compounds for the therapy of patients affected by this tumor. Yet, first reports on the use of these bioactive agents provided disappointing results. We therefore hypothesized that loss of membrane-bound TRAIL-R might be a feature of some CRC and that the evaluation of membrane staining rather than that of the overall expression of TRAIL-R might predict the response to TRAIL-R targeting compounds in this tumor.

**Aim and Methods:**

Thus, we evaluated the immunofluorescence pattern of TRAIL-receptors and E-cadherin to assess the fraction of membrane-bound TRAIL-receptors in 231 selected patients with early-stage CRC undergoing surgical treatment only. Moreover, we investigated whether membrane staining for TRAIL-receptors as well as the presence of KRAS mutations or of microsatellite instability (MSI) had an effect on survival and thus a prognostic effect.

**Results:**

As expected, almost all CRC samples stained positive for TRAIL-R1 and 2. Instead, membrane staining for these receptors was positive in only 71% and 16% of samples respectively. No correlation between KRAS mutation status or MSI-phenotype and prognosis could be detected. TRAIL-R1 staining intensity correlated with survival in univariate analysis, but only membranous staining of TRAIL-R1 and TRAIL-R2 on cell membranes was an independent predictor of survival (cox multivariate analysis: TRAIL-R1: p = 0.019, RR 2.06[1.12–3.77]; TRAIL-R2: p = 0.033, RR 3.63[1.11–11.84]).

**Conclusions:**

In contrast to the current assumptions, loss of membrane staining for TRAIL-receptors is a common feature of early stage CRC which supersedes the prognostic significance of their staining intensity. Failure to achieve therapeutic effects in recent clinical trials using TRAIL-receptors targeting compounds might be due to insufficient selection of patients bearing tumors with membrane-bound TRAIL-receptors.

## Introduction

Colorectal carcinoma (CRC) is a common malignancy accounting for over 1 million tumor cases worldwide and representing the fourth tumor-related cause of death. Unfortunately, while curative surgical therapies are feasible in patients with tumors in initial stage, the prognosis of patients with advanced disease remains disappointing [1].

In recent years, the advances in the understanding of the biology of CRC have led to the establishment of several mechanism-based therapies. Following the recognition of e.g. the role of EGF-R and VEGF-R in proliferation and angiogenesis, several compounds like cetuximab, panitumumab or bevacizumab have undergone clinical investigation and were shown to positively affect patients' survival [2]–[4]. It is expected that a comprehensive inventory of the contribution given by single signaling pathways to carcinogenesis will allow the employment of therapies tailored to a limited number of individual molecular targets.

Another recent instance of a mechanism-based therapy is represented by the development of compounds targeting the “death-receptors” TRAIL-R1 and TRAIL-R2 to selectively induce apoptosis in cancer cells [5]. The development of such agents is based on the rationale provided by studies showing that knocking out of TRAIL or blockage of TRAIL-receptors leads to enhanced tumor and metastasis formation *in vivo* [6] and that loss of TRAIL-receptors expression in human cancer tissues correlates with poor prognosis and tumor recurrence (Reviewed by Walczak and colleagues [7]). In this regard, we could recently show that membrane staining for TRAIL-receptors determines the prognosis of patients affected by hepatocellular carcinoma [8] and that the expression of the TRAIL-binding soluble decoy receptor OPG correlates with tumor stage and metastasis formation in patients affected by colon carcinoma [9]. At the present several TRAIL-receptors targeting compounds are undergoing clinical investigation in different tumor entities [5], [7].

Previous retrospective studies showing an almost invariable staining for TRAIL-receptors in colorectal cancer samples represented the rationale for the employment of TRAIL-receptors targeting agents in the treatment of this tumor. Unexpectedly however, the quantitative assessment of TRAIL-receptors staining intensity was associated with different prognostic outcomes in these studies [10]–[13]. In addition, first reports on early phase clinical trials with TRAIL-receptors targeting compounds in CRC showed disappointing results, prompting further investigation on a possible role of the receptors for TRAIL as therapeutic target in this tumor.

To address this problem we investigated a cohort of patients with early stage colon cancer with no nodal or distant metastasis undergoing no other treatment than surgery, and categorized tumor samples according to the presence or absence of TRAIL-receptors on the surface of tumor cells as an alternative to the sole semiquantitative assessment of TRAIL-receptors staining employed in previous studies. We found that colorectal cancers show a heterogeneous expression pattern of TRAIL receptor-1 and -2 with respect to their membranous occurrence. Differences in the expression of TRAIL-receptors in different subcellular compartments, rather than their staining intensity independently predicted the prognosis of CRC patients, thus representing a marker identifying a subset of tumors which have lost sensitivity to receptor-mediated apoptosis.

## Materials and Methods

### Clinical samples

Colorectal cancer specimens from patients who underwent surgical resection with curative intention between 1994 and 2004 at the University of Munich were retrieved from the archives of the institute of Pathology of our university. Collection of samples and of patients' information was conducted in anonymized form in agreement to the guidelines of the ethical committee of the University of Munich. Only colorectal adenocarcinomas with moderate differentiation (G2 according to the WHO classification), T-categories T2 and T3 having neither nodal (N0) nor distant metastasis (M0) at the time of diagnosis, and thus in stage I and IIA according to the TNM classification of colon cancer, were considered (T2/T3N0M0 G2) [14]. Furthermore, to minimize a possible influence of radio- or chemo-therapy on TRAIL-receptors status [15], [16] and on patients' prognosis, patients who underwent neoadjuvant or adjuvant therapy in addition to surgical treatment were excluded from this cohort. Survival data were retrieved from the tumor registry Munich (www.tumoregister-muenchen.de). Cases were censored where patients were lost to observation or died due to other reasons than colorectal cancer. The study complied with the requirements of the Ethics Committee of the Ludwig-Maximilian Universität of Munich.

### Construction of tissue microarrays

Colorectal tissue microarrays (TMA) were constructed as described previously [17]. Briefly 5 µm H&E stained sections of formalin fixed paraffin embedded (FFPE) tumor samples were used to define representative areas of viable tumor tissue. From these areas 1.0 mm diameter needle core-biopsies were taken from corresponding areas on the FFPE tumor blocks using a tissue arrayer (Beecher Instruments, Sun Prarie, WI, U.S.A.). The cores were placed in recipient paraffin array blocks at defined coordinates. To ensure that representative parts of the tumors were investigated three cores of each tumor were taken. To take also tumor heterogeneity into account, cores were taken from central tumor areas as well as from the invasive front. The cores in the paraffin block were incubated for 30 min at 37°C to improve adhesion between cores and paraffin of the recipient block.

### Immunohistochemistry

Immunohistochemical staining was done on 5 µm sections of TMA blocks. Anti-TRAIL-R1 monoclonal goat antibody (Santa Cruz Biotechnology Inc., Heidelberg, Germany Cat.No. sc-6823), Anti-TRAIL-R2 monoclonal rabbit antibody (Calbiochem, California, U.S.A. Cat.No. PC392), E-cadherin monoclonal mouse antibody (Invitrogen, Carlsbad, CA) were applied as primary antibody. Coimmunofluorescence was performed using the following fluoresceine labeled secondary antibodies: for TRAIL-receptors FITC-conjugated anti-goat IgG (Jackson Immuno Research laboratories, West Grove, PA) and for CDH1 a Cy3-conjugated anti-mouse IgG (Jackson Immuno Research Laboratories, West Grove, PA). These antibodies were previously used and validated [8]. Antigen retrieval was done by boiling the sections in Target Retrieval Solution (Dako, Hamburg, Germany) using a microwave oven 2 times each 15 min at 750 W. Endogenous peroxidase was blocked by incubation in 7.5% hydrogen peroxide for 10 minutes. Vectastain ABC-Kit Elite Universal (Vector Laboratories, CA, USA) together with AEC chromogen (Zytomed Systems) were used for development. Finally, slides were counterstained with hematoxylin (Vector).

### Evaluation of TRAIL-R1 and TRAIL-R2 immunohistochemistry

**Figure 1:**
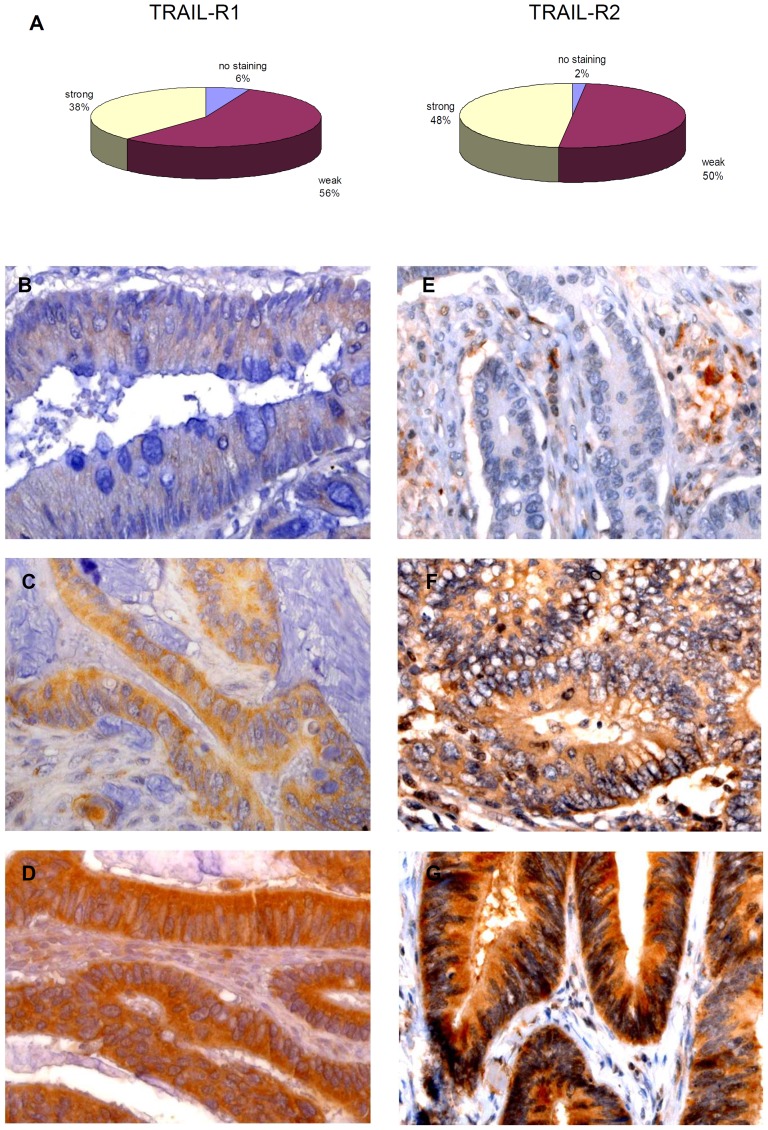
Semiquantitative evaluation of TRAIL-R1 and TRAIL-R2 staining in CRC cells. (A) Percentage of samples showing no staining, weak or strong immunoreactivity. (B–D): representative typical microscopic appearance of TRAIL-R1 staining (B: no staining. C: weak and D: strong staining). (D to F): staining of TRAIL-R2 (E: no staining. F: weak and G: strong staining). The present sections are representative of a grade 2 colonic cancer in stage II (T3N0M0) at the magnification of 630×.

**Figure 2:**
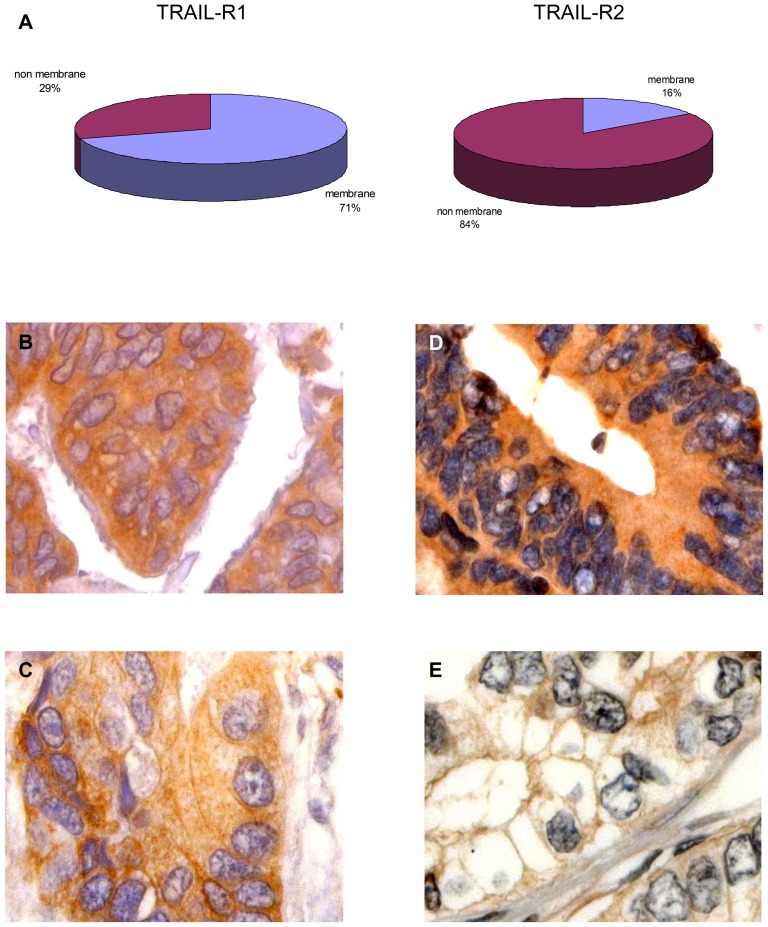
TRAIL-R1 and TRAIL-R2 staining in human colorectal cancer. (A) percentage of tumor samples showing membrane staining for TRAIL-R1 and TRAIL-R2. Representative typical microscopic appearance of TRAIL-R1 staining with predominant cytoplasmatic (B) or membrane staining (C). Typical pattern of TRAIL-R2 staining with predominant cytoplasmatic staining (D) or membrane staining (E). Magnification, ×800. The present sections are representative of a grade 2 colonic cancer in stage II (T3N0M0) at the magnification of 630×.

**Figure 3:**
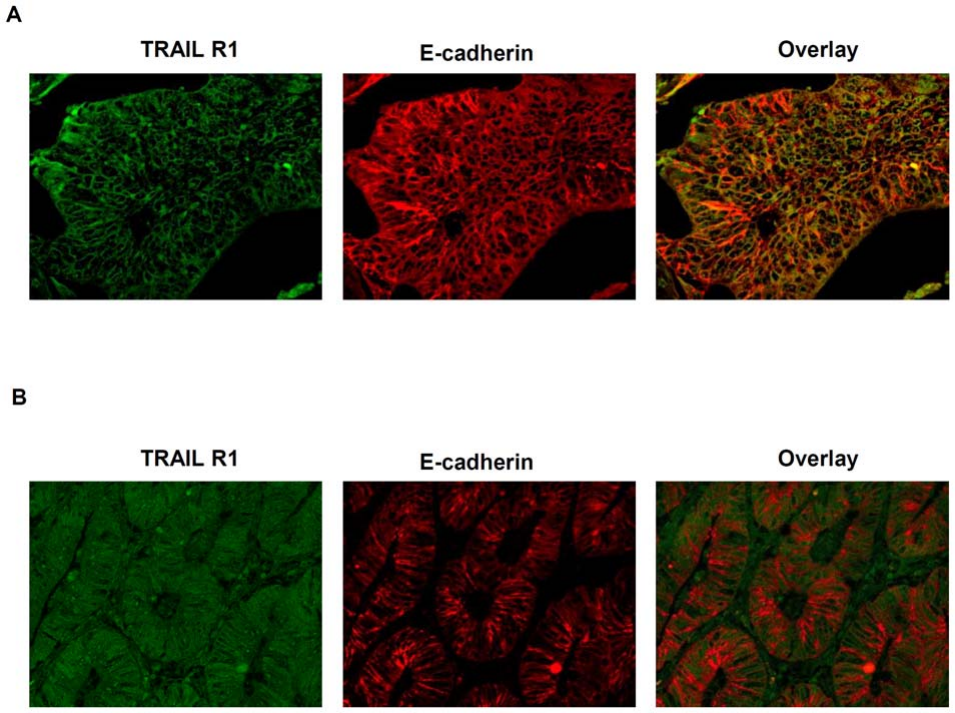
Membranous localization of TRAIL-receptors and E-cadherin. Representative pattern of co-staining of TRAIL-R1 and E-Cadherin on cell membranes of colorectal cancer cells by confocal microscopy showing (A) a pattern of predominant membrane staining vs. (B) non membranous staining. Staining for TRAIL-R1 (green, left panel), E-cadherin (red, middle panel) and overlays of these staining (right panel).

TRAIL-R1 and TRAIL-R2 immunostaining was evaluated by grading the staining intensity according to a semiquantitative score ranging from 0 to 2, respectively for negative, weak and strong positive degrees of immunoreactivity (Figure 1). According to the rationale that a prerequisite for functional activity of TRAIL-receptors is the membranous surface expression [18], a second evaluation was done by categorizing tumor samples according to the presence or absence of TRAIL-receptors staining on cell membranes regardless of the concomitant presence of cytoplasmatic staining and its staining intensity (Figure 2). In addition to the inspection at conventional light microscopy, to more sensitively discriminate between membrane and cytoplasmatic staining for TRAIL-receptors, the immunofluorescence pattern of co-staining of TRAIL-receptors and E-cadherin was performed by using confocal microscopy (representative staining patterns in Figure 3). Images were captured with an LSM 700 device (Zeiss) using a Plan Apochromat 20×/0.8 M27 objective, ZEN 2009 software (Zeiss) and the following settings: image size 2048×2048 and 16 bit; pixel/dwell of 25.2 µs; pixel Size 0.31 µm; laser power 2%; master gain 600–1000. After image capturing the original LSM files were converted into TIFF files. To exclude intraobserver variability specimens were evaluated twice by an observer who had no prior knowledge of prognosis or other clinic-pathological variables. Exemplary features of TRAIL-receptors staining in cancer samples or in non-tumor colonic tissues are respectively shown in Figure 1, Figure 2, and in Figure S1.

### Analyses of KRAS mutations

For the analyses of mutations in exon 2 of the KRAS oncogene in codon 12/13, material was left from only 200 of the 231 patients (86.6%). Therefore, genomic DNA was extracted from microdissection of tissue areas containing tumor as previously described [19]. Pyro-sequencing was done using the Pyro-Gold kit (Qiagen, Germany) and HotStar Taq-Polymerase (Qiagen, Germany). The PF2 primer was used to determine anti-sense sequences. The PyroMark Q24 device (Qiagen, Germany) and the PyroMark™ Q24 software were used for sequencing, and sequence analyses [20], [21].

### Microsatellite stability analysis

To determine the status of microsatellite stability [microsatellite stability (MSS) or high-grade microsatellite instability (MSIH)], two mononucleotide repeat markers BAT-25 and BAT-26 were investigated. DNA was amplified in a duplex PCR (Qiagen DNA Multiplex PCR kit, 100 nM BAT25 and 100 nM BAT26-specific primers – Table S3) applying the following cycle profile: denaturation at 95°C for 15 min, 34 cycles of denaturation at 94°C for 30 sec, annealing at 57°C for 90 sec and extension at 72°C for 60 sec, with a final extension step at 60°C for 30 min, as previously described [22], [23]. 1 µl of the PCR product was mixed with 18.5 µl of highly deionized formamide (HiDi formamide) and 0.5 µl DNA Size Standard LIZ 500 / (−250) (both Applied Biosystems, Darmstadt, Germany). This mixture was denaturated for 3 min at 94°C, immediately put on ice, and separated using an ABI 3130 Genetic Analyzer. Results were analysed using GeneMapper Software (Applied Biosystems).

### Analysis of the expression of the splice forms KRAS4A and KRAS4B

For this investigation sufficient material was left for only 128 of 231 tumor samples (55.4%). Therefore whole RNA was isolated from microdissected tumor areas using RNeasy kits (Qiagen; 74404) as previously described [24]. RNA concentrations were measured by UV-photometry. 200–1000 ng of RNA isolates were reverse transcribed in the presence of 100 µM random hexamer primers and of 200 U RevertAid Reverse Transcriptase (both by Fermentas, St. Leon, Germany; SO142, EP0441). 2 µl of the crude RT-reaction were used as the template in RT-qPCRs employing Light Cycler 480 Probes Master (Roche; 04902343001) with specific primer-pairs and Universal ProbeLibrary Probes (Roche – Table S3). Cp (critical point) values of RT-qPCRs specific for *KRAS4A, KRAS4B* and the reference gene *HPRT* (*hypo-xanthin phosphoribosyl- transferase*) were determined employing a LightCycler 480 device (Roche). All concentrations of *KRAS4A, KRAS4B* -specific RNAs were normalized on the expression of the *HPRT* gene (ΔCp). Experiments were done in duplicates and repeated at least twice. To validate the experimental system, relative amounts of the two splice variants KRAS4A and KRAS4B were assessed in cell lines SW948 and HCT15 as it had been described that SW948 express more KRAS4A variant than HCT15 cells [25]. This result was reproducible (Figure S3) thus validating the experimental set-up. Moreover, our read out system demonstrated high robustness as calibration curves using defined amounts of template DNA showed linearity at least over four log scales down to 100 copies of the specific type of RNA (Figure S3).

### Statistical analysis

Cross-tabulations were calculated using Fisher's exact test. Kaplan-Meier analysis was used to estimate cancer specific survival. Significance of the Kaplan-Meier statistic was tested applying the log-rank test. Multivariate analysis was done by using the multivariate Cox regression model. Statistics were calculated using SPSS version 15.0 (SPSS Inc.). p-values<0.05 were considered to be statistically significant.

## Results

### Patients and samples

The screening for cancer specimens was conducted in patients who underwent surgical resection with curative intention between 1994 and 2004 at our institution. The selection of samples was limited to colorectal adenocarcinomas with moderate differentiation (G2), T-categories T2 and T3 in patients having neither nodal (N0) nor distant metastasis (M0) at the time of diagnosis, and limited to patients receiving surgical treatment only. This resulted in a collection of 231 patients for the analysis. The collection consisted of 55% male and 45% female patients. 64% of the patients were older than 65 years (mean age 56±8.1 years) while the remaining 36% of patient had a mean age of 75±6.9 years. 85% of patients were diagnosed with a tumor in stage T3 while 15% of patients were affected by a tumor in stage T2. The survival data was censored as case follow up was discontinued or patients died for other reasons than colorectal cancer. The characteristics of this patient population are summarized in Table 1.

**Table 1:** Clinical and pathological characteristics of CRC patients.

Variable	N (%)
Gender	
Male	126 (55%)
Female	105 (45%)
Age, y	
<65	84 (36%)
≥65	147 (64%)
T-category	
T2	34 (15%)
T3	197 (85%)
KRAS status	
No mutation	74 (37%)
Mutation	126 (63%)
MSI-phenotype	
Instable	69 (35%)
Stabie	126 (65%)

### TRAIL-R1 and TRAIL-R2 staining in colorectal cancer samples

To assess the staining of TRAIL-receptors in tumor samples we first performed a semi-quantitative analysis based on the categorization of samples according to the absence of staining, or the presence of a weak or strong staining intensity for the respective receptors. According to this criterion, 87 (38%) of colon cancer samples showed a strong positive staining, 129 (56%) showed a weak staining, whereas 15 (6%) samples stained altogether negative for TRAIL-R1 (Figure 1). As TRAIL-R2 in tumor samples was examined, a strong staining was observed in 110 (48%) of cases, a weak staining in 116 (50%), while only 5 (2%) samples stained negative for TRAIL-R2 (Figure 1). In a subsequent analysis we determined the cellular distribution of TRAIL-R1 and TRAIL-R2 by categorizing tumor samples according to the presence or absence of staining for the respective TRAIL-receptors on the cell membranes. As judged by immunohistochemical staining and the overlapping immunofluorescence pattern of TRAIL-receptors and of E-cadherin, membrane staining for TRAIL-R1 could be detected in 163 (71%) cases, whereas negative staining or exclusively cytoplasmatic staining was found in 68 (29%) cases. For TRAIL-R2 membrane staining was observed in 36 cases (16%); cytoplasmatic or negative staining was observed in 195 cases (84%).

Therefore, while most tumor samples stained altogether positive for TRAIL-receptor 1 and 2, the fraction of tumor samples showing membrane staining for these receptors was considerably lower. When the expression intensity and the cellular distribution of TRAIL-receptors on tumor samples were analyzed in relation to different clinico-pathological variables including KRAS-mutation status and the presence of microsatellite instability, no correlation could be detected as judged by Fisher's exact test (Tables S1 and S2).

### Prognostic significance of TRAIL-receptor expression and cellular localization in colorectal cancer

**Figure 4:**
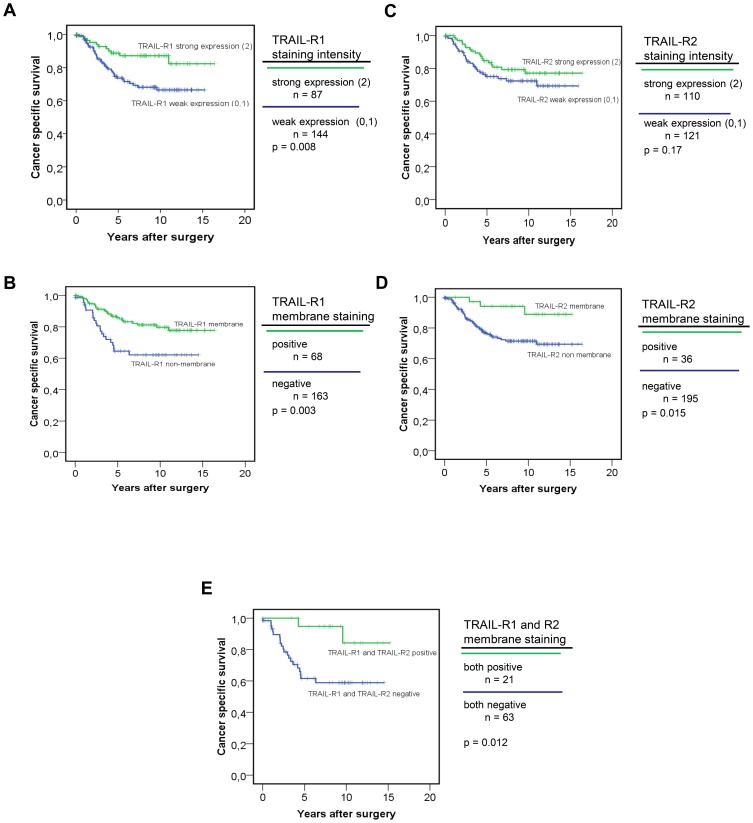
TRAIL-R1 staining and survival. (**A**) Survival plot of patients affected by colorectal cancer acc. to TRAIL-R1 staining intensity. In this and the following graphs censored cases are indicated by a cross. (B) survival curves of the same patients' population categorized according to TRAIL-R1 staining on cell membrane. (C) Survival plot of patients according to the staining intensity for TRAIL-R2 (strong vs. weak expression). (D) survival plot of the same patient's population categorized according to TRAIL-R2 cellular distribution. (E) Survival of patients according to membrane staining status of both TRAIL-receptors. Kaplan-Meier curves represent overall survival related to membrane staining of TRAIL receptor 1 and 2 vs. patients bearing tumors staining negative for both TRAIL receptors.

When the expression of TRAIL-receptors was considered in relation to the survival of colorectal cancer patients, TRAIL-R1 staining intensity (high expression vs. low/no expression) was associated with a significantly better prognosis: the 5-year survival of patients bearing tumors with overall higher TRAIL-R1 expression was 70% vs. 56% of patients with low or altogether no staining for TRAIL-R1; the 10-year survival for these patients was respectively 31% vs. 25% (p = 0.008; Figure 4A). Additionally, when tumor samples were categorized according to the presence or absence of membrane staining for TRAIL-receptors, patients with tumors exhibiting TRAIL-R1 staining on the surface of cell membranes were shown to have a better prognosis vs. patients with cytoplasmatic or no staining (5- year survival 65% vs. 44%; 10-year survival: 30% vs. 22%, p = 0.003 – Figure 4B).

When TRAIL-R2 staining was considered, its intensity of expression did not significantly correlate with survival (p = 0.17; Figure 4C). However, if patients were stratified according to the presence or absence of staining on the cell membranes, membrane staining for TRAIL-R2 in tumor samples correlated with a significantly better patient survival (5-year survival: 83% vs. 57%; 10-year survival: 38% vs. 26% p = 0.015; Figure 4D). When patients' survival was analyzed according to the double positivity for TRAIL-receptors, i.e. when the survival of patients with simultaneous membrane staining for TRAIL-R1 and TRAIL-R2 was compared to that of patients exhibiting no staining or cytoplasmatic staining only, the survival of patients with membrane staining for both TRAIL-receptors increased to 92% vs. 44% of patients with no membranous staining (p = 0.012, Figure 4E). A further analysis considering the possibly that, as previously reported, KRAS mutations, a decreased amount of the splicing variant KRAS4A relative to the splice variant KRAS4B, or an MSI-phenotype might influence the survival of colorectal cancer patients [26]–[28], failed to show any significant prognostic effect in our patients' cohort (Figure S2).

Finally, to assess the specific influence of TRAIL-receptors staining on survival, a Cox regression analysis including simultaneously the cellular distribution of TRAIL-receptors, their staining intensity and other relevant clinico-pathological variables was performed. This showed that membrane staining for TRAIL-R1 and for TRAIL-R2 individually and independently predicts the survival of our colorectal cancer patients collective (TRAIL-R1: p = 0.019, RR 2.06 [1.12–3.77]; TRAIL-R2: p = 0.033, RR 3.63 [1.11–11.84]). In contrast, in spite of the significant association found in the long-rank test, the staining intensity/expression of TRAIL-receptors could not be confirmed as independent risk factor for recurrence (Table 2).

**Table 2:** Multivariate survival analysis including TRAIL-R1 and TRAIL-R2 membrane staining, staining intensity and relevant clinico-pathological variables.

Variable		Relative risk	p
		(95% confidence interval)	
TRAIL-R1 membrane staining			
Positive	162 (70%)	1.00	
Negative	69 (30%)	2.06 (1.12–3.77)	**0.019**
TRAIL-R2 membrane staining			
Negative	204 (88%)	1.00	
Positive	27 (12%)	3.63 (1.11–11.84)	**0.033**
TRAIL-R1 staining intensity			
Strong staining	87 (38%)	1.00	
Weak/no staining	144 (62%)	1.62(0.65–4.05)	0.302
Gender			
Male	126 (55%)	1.00	
Female	105 (45%)	0.88 (0.49–1.56)	0.651
Age, y			
<65	84 (36%)	1.00	
≥65	147 (64%)	1.20 (0.65–2.20)	0.555
T-category			
T2	34 (15%)	1.00	
T3	197 (85%)	1.07 (0.488–2.35)	0.865

## Discussion

### TRAIL-receptors in the pathophysiology and therapy of colorectal cancer

The loss of TRAIL-receptors has been shown to play an important role in cancer development. In particular, several different studies support the notion that TRAIL signaling plays *in vivo* an important function in preventing metastasis formation [29]–[32]. Recently it was also shown that the expression of TRAIL-receptors correlates with that of several markers of apoptosis thus providing a link between the functional role of these receptors and their prognostic significance [33].

Previous reports on the almost ubiquitous expression of TRAIL-receptors in CRC represented the rationale for the use of TRAIL-receptors targeting agents for the treatment of this tumor. Surprisingly however, while the frequent loss of TRAIL-receptors reported for several tumor entities might represent an obstacle to the clinical efficacy of such compounds [8], [10], [34], [35] no systematic evaluation of membrane staining of TRAIL-receptors in CRC samples is available. Furthermore, first reports on early phase clinical trials with TRAIL-receptors targeting agonistic antibodies in CRC failed to show clear signs of efficacy [36] prompting further investigation on TRAIL-receptors as therapeutic target in the treatment of this tumor. To address this issue, basing on our recent findings in hepatocellular carcinoma [8] we adopted the evaluation of the cellular distribution of TRAIL-receptors as criterion for evaluating their prognostic significance. Also, to reduce potential biasing factors, we decided to analyze a homogenous patient collective with tumors in early stage with no metastasis undergoing surgery only.

### Prognostic relevance of TRAIL-receptors staining intensity

In agreement with previous studies [10], [12], [13], in our cohort the vast majority of samples showed positive staining for TRAIL-R1 and TRAIL-R2, roughly half of samples showing a strong staining (Figure 1). As we assessed the prognostic significance of TRAIL-receptors staining, TRAIL-R1 intensity staining scores (strong vs. low/no-staining) showed a significant correlation with survival in the long-rank test, higher TRAIL-R1 staining intensity being associated with better survival (p = 0.008, Figure 4A); in contrast, TRAIL-R2 staining intensity, KRAS-status, the relative amount of the splice variant KRAS4A or MSI-phenotype showed no correlation with survival (Figure 4C, Figure S2). These results are in agreement with previous studies which reported a positive correlation between patients' survival and expression of TRAIL-R1 [10], [33]. For unknown reasons, no effect [13] or even a negative correlation for TRAIL-R1 but trendy positive correlation for TRAIL-R2 with survival were also reported [12].

### Prognostic relevance of the cellular distribution of TRAIL-receptors as alternative to staining intensity

In the attempt to further clarify this issue, we subsequently evaluated whether the presence or absence of membrane staining of TRAIL-receptors could better correlate with survival then the sole evaluation of their staining intensity. In contrast to the almost ubiquitous staining for TRAIL-receptors, a considerable fraction of samples showed negative membrane staining for TRAIL-R1 and TRAIL-R2 (Figure 2). By adopting this criterion, five-year survival in patients bearing tumors exhibiting TRAIL-R1 or TRAIL-R2 staining on cell membranes was higher than that of patients showing no staining or cytoplasmatic staining only (Figure 4). Patients bearing tumors with double-positive membrane staining for both TRAIL-receptors survived significantly longer in comparison to patients showing double-negative membrane staining (Figure 4E). The fact that in the multivariate analysis comprising the effect of the cellular distribution of TRAIL-receptors as well as that of their staining intensity, the latter could not be confirmed as independent prognostic factor suggests that the detection of TRAIL-receptors staining on cell membrane is the major determinant of survival: this is consistent with the data available for patients affected by hepatocellular carcinoma [8], with recent *in vitro* evidence on the role of TRAIL-receptors internalization in the resistance to TRAIL [18], and with the notion that membrane-bound TRAIL-receptors are exposed to the effect of circulating TRAIL. Previous studies had shown no prognostic significance for TRAIL-R2, or only a trend toward a positive correlation between the expression of this receptor and survival [12]; we hypothesize that failure to recognize the role of TRAIL-R2 in determining patients' survival in previous studies reflects the higher prognostic significance of the cellular distribution of TRAIL-receptors vs. that of their staining intensity.

### Clinical consequences of the functional meaning of TRAIL-receptors staining on cell membranes

Loss of expression of TRAIL-receptors has potential consequences regarding the employment of agonistic antibodies targeting TRAIL-receptors at this time undergoing clinical investigation as cancer therapy: although we could confirm that the vast majority of tumors stained positive for TRAIL-receptors, we found that loss of TRAIL-receptors on the cell membrane is a frequent feature of CRC with predominant prognostic significance; it should be therefore considered whether failure to show signs of efficacy in recent clinical trials using TRAIL-receptors agonistic antibodies [36] might be attributable to insufficient selection of patients bearing tumors with membrane-bound TRAIL-receptors. On the other hand, due to the summation of the prognostic effects of TRAIL-R1 and TRAIL-R2, patients exhibiting membrane staining for both receptors might profit by the combined administration of antibodies targeting both receptors or by the administration of recombinant TRAIL [37].

The fact that, independently of their cellular localization, almost all tumor samples showed some extent of staining for TRAIL-receptors is in agreement with the notion that genetic loss or mutation of TRAIL-receptors is a rare event in cancer cells [34], [38]. Differences in the cellular distribution of these receptors suggests instead that impairment of TRAIL-receptors trafficking to the outer cell membrane or mechanisms of internalization play a role in determining the functional loss of TRAIL-receptors. To this regard, endocytosis mediated by clathrin was recently described as cause of resistance to TRAIL in breast cancer cells [18] and several compounds were shown to increase expression of TRAIL-receptors as well as their localization onto the cell membranes [39], [40]. Internalization of TRAIL-receptors seems to be therefore a potentially reversible cause for resistance to TRAIL and administration of such compounds might enhance apoptosis induction in combination with TRAIL-receptors targeting agents [39].

### Summary

We propose the analysis of membrane staining for TRAIL-receptors as prognostic marker in early stage colorectal cancer and as possible biomarker of response to TRAIL-receptors targeting agents. Prospectively collected data based on the efficacy of these compounds will provide a definitive answer to this issue.

## Supporting Information

Figure S1 **Typical pattern of TRAIL-receptor staining in normal colonic mucosa showing strong (A,C) or weak (B,D) staining for TRAIL-R1 and TRAIL-R2 (Original magnification 630×).**
(TIF)

Figure S2 **Survival plot of patients affected by colorectal cancer acc. to: (A) KRAS status, (B) microsatellite status (MSI = microsatellite instability; MSS = microsatellite stability), (C) amount of KRAS4A splice variant relative to KRAS4B. Censored cases are indicated by a cross.**
(TIF)

Figure S3 **Validation of RT-qPCR based assessment of KRAS4A and KRAS4B splicing variants.** To assess the validity and efficiency of quantitative PCR analysis performed in samples isolated from our tissue-specimens, splicing variant KRAS4A was assessed in cell lines SW948 and HCT15 as previous immunohistochemical evaluation of these cell lines showed the maximal differential expression of this splicing variant of KRAS between these two cell lines (Abubaker et al. J Pathol 2009; 219: 435–445). As shown in panel A, accordingly to this previous report, KRAS4A production was about sevenfold higher in SW948 than in HCT15 cells. Moreover, the dynamic range of the measurement was very high as the calibration curves displayed linear slopes and linearity was granted for at least four log-scales down to 100 molecules (panel B).
(TIF)

Table S1 **Correlation between TRAIL-receptors staining intensity and clinico-pathological variables in tumor samples.**
(PDF)

Table S2 **Correlation between membrane staining of TRAIL-receptors and clinico-pathological variables in tumor samples.**
(PDF)

Table S3 **PCR primers.**
(DOC)
