# Supplementary figures and images for: Microsatellite Instability, KRAS Mutations and Cellular Distribution of TRAIL-Receptors in Early Stage Colorectal Cancer
Source: PLoS One. 2012 Dec 20;7(12):e51654. doi: 10.1371/journal.pone.0051654 (PMC3527471; doi:10.1371/journal.pone.0051654)

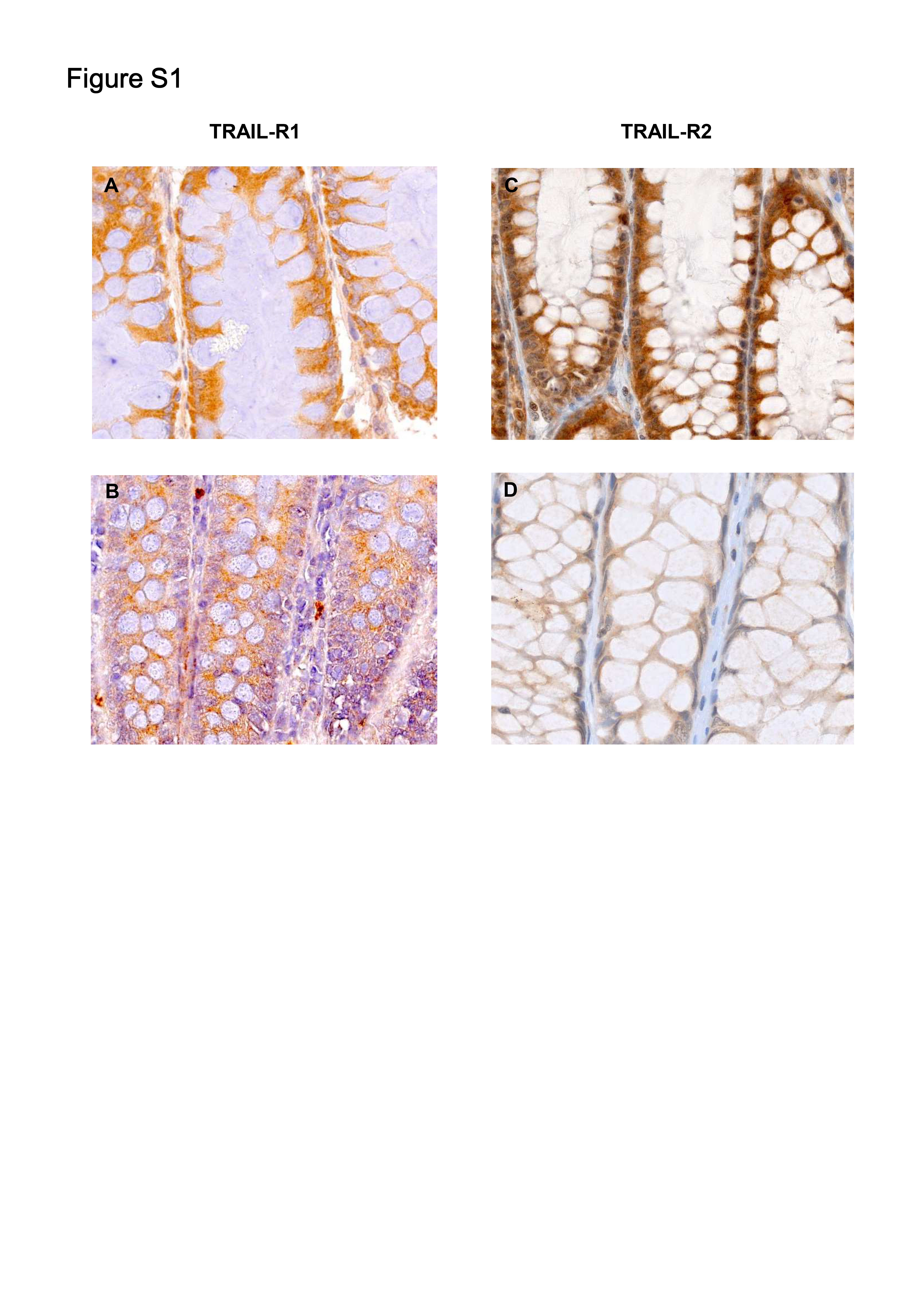

Supplement: Figure S1 — Typical pattern of TRAIL-receptor staining in normal colonic mucosa showing strong (A,C) or weak (B,D) staining for TRAIL-R1 and TRAIL-R2 (Original magnification 630×). (TIF) [file pone.0051654.s001.tif]

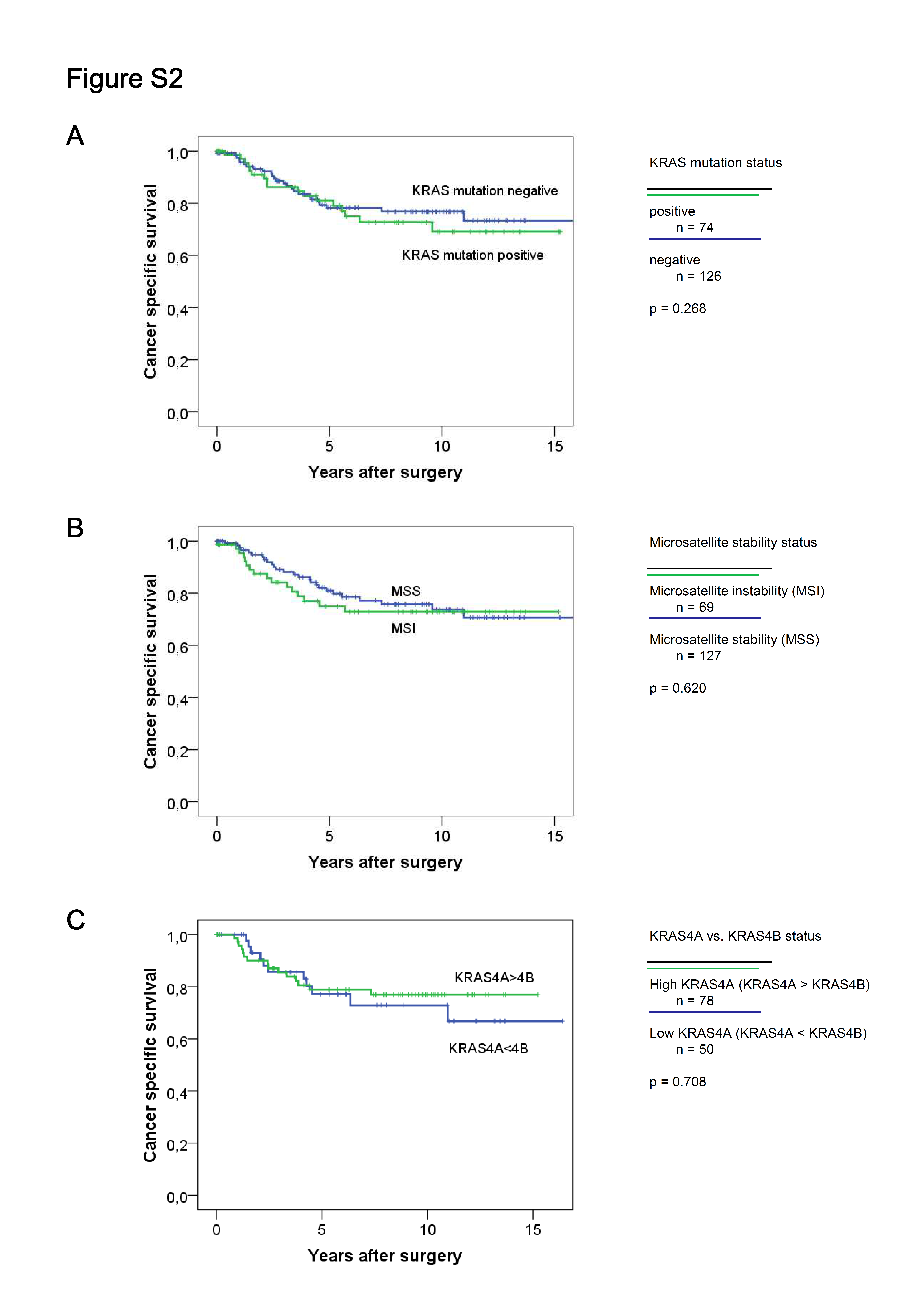

Supplement: Figure S2 — Survival plot of patients affected by colorectal cancer acc. to: (A) KRAS status, (B) microsatellite status (MSI = microsatellite instability; MSS = microsatellite stability), (C) amount of KRAS4A splice variant relative to KRAS4B. Censored cases are indicated by a cross. (TIF) [file pone.0051654.s002.tif]
