# Supplementary material for: Microsatellite Instability, KRAS Mutations and Cellular Distribution of TRAIL-Receptors in Early Stage Colorectal Cancer
Source: PLoS One. 2012 Dec 20;7(12):e51654. doi: 10.1371/journal.pone.0051654 (PMC3527471; doi:10.1371/journal.pone.0051654)
Supplement: Table S1 — Correlation between TRAIL-receptors staining intensity and clinico-pathological variables in tumor samples. (PDF) [file pone.0051654.s004.pdf]

	TRAIL-R1				TRAIL-R2		
**Variable**	**weak/no staining**	**strong staining**	**p**	**Variable**	**weak/no staining**	**strong staining**	**p**
Gender				Gender			
Male	83	44	0.14	Male	68	58	0.6
Female	61	43		Female	53	52	
Age, (years)				Age, (years)			
< 65	51	33	0.40	< 65	49	35	0.17
>= 65	93	54		>= 65	72	75	
T-category				T-category			
T2	20	14	0.39	T2	23	11	0.06
T3	124	73		T3	98	99	
KRAS				KRAS			
No mutation	78	48	0.28	No mutation	72	54	0.32
Mutation	42	32		Mutation	39	35	
MSI-phenotype				MSI-phenotype			
Instable	42	27	0.53	Instable	53	33	0.25
Stabie	78	49		Stabie	74	36	

Additional Table 1: correlation between TRAIL-receptors staining intensity and clinico-pathological variables in tumor samples
