# Supplementary material for: Microsatellite Instability, KRAS Mutations and Cellular Distribution of TRAIL-Receptors in Early Stage Colorectal Cancer
Source: PLoS One. 2012 Dec 20;7(12):e51654. doi: 10.1371/journal.pone.0051654 (PMC3527471; doi:10.1371/journal.pone.0051654)
Supplement: Table S2 — Correlation between membrane staining of TRAIL-receptors and clinico-pathological variables in tumor samples. (PDF) [file pone.0051654.s005.pdf]

Variable	TRAIL-R1 membrane	TRAIL-R1 cytoplasm	p	Variable	TRAIL-R2 membrane	TRAIL-R2 cytoplasm	p
Gender				Gender			
Male	88	38	0.89	Male	21	105	0.72
Female	75	30		Female	15	90	
Age, (years)				Age, (years)			
< 65	64	20	0.23	< 65	18	66	0.06
>= 65	100	47		>= 65	17	129	
T-category				T-category			
T2	25	9	0.84	T2	2	34	0.12
T3	138	59		T3	25	163	
KRAS				KRAS			
No mutation	84	42	0.12	No mutation	15	111	0.08
Mutation	56	18		Mutation	15	59	
MSI-phenotype				MSI-phenotype			
Instable	50	19	0.26	Instable	7	62	0.13
Stabie	85	42		Stabie	22	105	

Additional Table 2: correlation between cellular distribution of TRAIL-receptors staining and clinico-pathological variables in tumor samples
