# Supplementary material for: Microsatellite Instability, KRAS Mutations and Cellular Distribution of TRAIL-Receptors in Early Stage Colorectal Cancer
Source: PLoS One. 2012 Dec 20;7(12):e51654. doi: 10.1371/journal.pone.0051654 (PMC3527471; doi:10.1371/journal.pone.0051654)
Supplement: Table S3 — PCR primers. (DOC) [file pone.0051654.s006.doc]

**Additional Table 3.**

| Name | Sequence | PCR product [bp] | conc | UPL no |
| --- | --- | --- | --- | --- |
| BAT25 | *FAM*-TCTGCATTTTAACTATGGCTC  TCGCCTCCAAGAATGTAAGT | 124 | 100 nM | - |
| BAT26 | *VIC*-AACCATTCAACATTTTTAACCC  TGACTACTTTTGACTTCAGCC | 121 | 100 nM | - |
| KRAS4A | GAAACATCAGCAAAGACAAGACA  TCGGATCTCCCTCACCAAT | 66 | 300nM  900nM | 88 |
| KRAS4B | TGGACGAATATGATCCAACAAT  TCCCTCATTGCACTGTACTCC | 121 | 300 nM  300 nM | 62 |
| HPRT | tgaccttgatttattttgcatacc  cgagcaagacgttcagtcct | 120 | 900 nM  900 nM | 73 |

Conc – concentration; FAM – 6-Fluorescein amidite; HPRT – hypo-xanthin-phospho-ribosyl-transferase (reference gene); no-number;  UPL – universal probe library (Roche®); VIC®- VIC-dye (Applied Biosystems)
